# Cryo-EM structure of a 40 kDa SAM-IV riboswitch RNA at 3.7 Å resolution

**DOI:** 10.1038/s41467-019-13494-7

**Published:** 2019-12-03

**Authors:** Kaiming Zhang, Shanshan Li, Kalli Kappel, Grigore Pintilie, Zhaoming Su, Tung-Chung Mou, Michael F. Schmid, Rhiju Das, Wah Chiu

**Affiliations:** 1 Department of Bioengineering, and James H. Clark Center, Stanford University, Stanford, CA 94305 USA; 2 Biophysics Program, Stanford University, Stanford, CA 94305 USA; 3 Center for Biomolecular Structure and Dynamics, University of Montana, Missoula, MT 59812 USA; 4 Division of CryoEM and Bioimaging, SSRL, SLAC National Accelerator Laboratory, Stanford University, Menlo Park, CA 94025 USA; 5 Department of Biochemistry, Stanford University, Stanford, CA 94305 USA; 6 Department of Physics, Stanford University, Stanford, CA 94305 USA

**Keywords:** RNA, Cryoelectron microscopy

## Abstract

Specimens below 50 kDa have generally been considered too small to be analyzed by single-particle cryo-electron microscopy (cryo-EM). The high flexibility of pure RNAs makes it difficult to obtain high-resolution structures by cryo-EM. In bacteria, riboswitches regulate sulfur metabolism through binding to the S-adenosylmethionine (SAM) ligand and offer compelling targets for new antibiotics. SAM-I, SAM-I/IV, and SAM-IV are the three most commonly found SAM riboswitches, but the structure of SAM-IV is still unknown. Here, we report the structures of apo and SAM-bound SAM-IV riboswitches (119-nt, ~40 kDa) to 3.7 Å and 4.1 Å resolution, respectively, using cryo-EM. The structures illustrate homologies in the ligand-binding core but distinct peripheral tertiary contacts in SAM-IV compared to SAM-I and SAM-I/IV. Our results demonstrate the feasibility of resolving small RNAs with enough detail to enable detection of their ligand-binding pockets and suggest that cryo-EM could play a role in structure-assisted drug design for RNA.

## Abstract (web summary)

The conformational heterogeneity of RNA molecules makes their structure determination by X-ray crystallography and NMR challenging. Here the authors show that RNA structures can be solved by cryo-EM and present the structures of a 40 kDa SAM-IV riboswitch in the apo form and bound to its ligand S-adenosylmethionine.

## Introduction

Recent technological breakthroughs in single-particle cryo-electron microscopy (cryo-EM) have resulted in numerous near-atomic or atomic resolution structures of biological molecules. To date, nearly all of the high-resolution structures determined by cryo-EM are proteins or protein-RNA complexes, with molecular weight larger than 50 kDa. For pure RNA specimens, cryo-EM has not been regarded as a useful technique for high-resolution structure determination due to presumed high conformational flexibility of small RNA-only structures. So far, only the HIV-1 dimerization initiation sequence (DIS, 94-nt) has been resolved at subnanometer resolution by cryo-EM^1^.

Metabolite-binding riboswitches are non-coding RNAs that bind to metabolites with high specificity and regulate downstream gene expression in response to changes in metabolite concentrations^2^. These molecules are of growing interest as potential targets for new classes of antibiotics^3–5^. S-adenosylmethionine (SAM), a cofactor used in many methylation reactions, is specifically recognized by seven classes of riboswitches, including SAM-I, SAM-II, SAM-III, SAM-IV, SAM-I/IV, SAM-V, and SAM-VI^6^. To date, the high-resolution structures of SAM-I^7^, SAM-II^8^, SAM-III^9^, SAM-I/IV^10^, and SAM-V^11^ have been obtained by X-ray crystallography, providing insights into their molecular recognition mechanisms. Although SAM-IV riboswitches were originally identified by bioinformatics more than a decade ago^12^, their tertiary structures have not yet been resolved.

Here, we use cryo-EM to determine maps of both apo and ligand-bound SAM-IV riboswitches (119-nt, ~40 kDa) from *Mycobacterium sp*. to the resolution of 3.7 Å and 4.1 Å, respectively. The resulting cryo-EM maps allow us to confidently build models with our recently implemented Rosetta modeling software, auto-DRRAFTER^13^. This study not only reveals the structural basis of ligand recognition by the SAM-IV riboswitch but also demonstrates the feasibility of cryo-EM for structure determination below the current size limit and detection of ligand-binding sites.

## Results

### Single-particle cryo-EM analysis

**Fig. 1:**
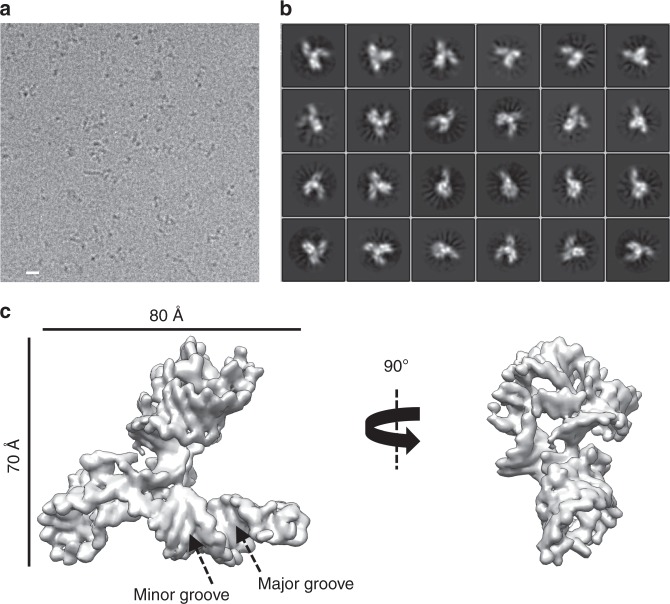
Single-particle cryo-EM analysis of the apo SAM-IV riboswitch collected on Titan Krios. **a** Representative motion-corrected cryo-EM micrograph. Scale bar represents 100 Å. **b** Reference-free 2D class averages computed in Relion. **c** Final 3D reconstruction in two different views.

To test the feasibility of using cryo-EM to determine small RNA structures, a small dataset of the apo SAM-IV riboswitch containing ~500 micrographs was first collected on a 200 keV Talos Arctica G2 Cryo-Transmission Electron Microscope (Thermo Fisher Scientific) equipped with a Gatan K2 Summit camera and the Volta phase plate applied to increase the image contrast. About 23,000 particles were selected for 2D classification and 3D reconstruction. Supplementary Fig. 1a shows the raw micrograph, where the particles appear as black spots without showing fine details due to the small molecular weight. The 2D averages of the riboswitch display a “three-way junction” shape and random particle orientation (Supplementary Fig. 1b). The final 3D map was refined to a resolution of ~7 Å, revealing the major grooves in the RNA duplex (Supplementary Fig. 1c, d). With this encouraging result, we explored eliminating the Volta Phase Plate to make the data collection simpler. Interestingly, a similar structure was obtained quite readily. We then decided to record a larger dataset collection on a 300 keV Titan Krios G3 Cryo-Transmission Electron Microscope (Thermo Fisher Scientific) equipped with a Gatan K2 Summit camera without phase plate. A total of ~7000 micrographs were used for further analysis. Compared to the small dataset, the resulting 2D averages and a 3.7-Å 3D reconstruction reveal more detailed RNA features (Fig. 1 and Supplementary Fig. 2), including all well-resolved major and minor grooves, a traceable sugar phosphate backbone, and some visible base pairs (Supplementary Movie 1). Supplementary Fig. 2d shows the linear correlation between the particle number and the map resolution^14^, and the B-factor is estimated to be ~219 Å^2^. It can be inferred from this plot that using a dataset of similar quality to obtain a 3-Å resolution map of this RNA would require more than 40 million particles, which is ~50 times the number of particles in this work.

In addition to this map of the RNA without ligand, a dataset of the SAM-bound state was also collected and processed using the same scheme as the apo state. The resulting 4.1-Å cryo-EM map displays visually similar structural features (Supplementary Fig. 3), allowing us to explore the possible ligand recognition mechanism of SAM-IV.

### Overall architecture of SAM-IV riboswitches

**Fig. 2:**
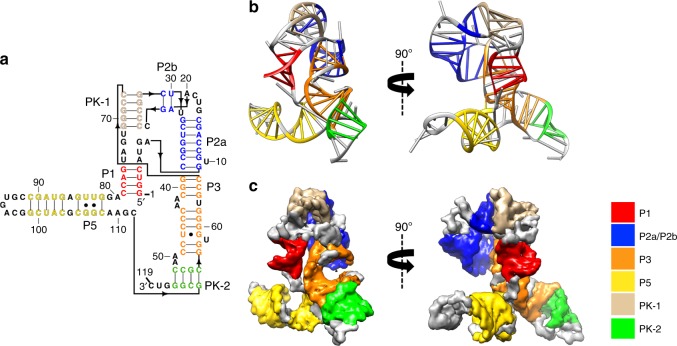
The secondary and tertiary structures of the apo SAM-IV riboswitch. **a** The secondary structure with the domains colored differently. The black arrow indicates the direction of the backbone. **b**-**c** The model and map colored by the same scheme as in **a**. P paired, PK pseudoknot.

At the attained ~4-Å map resolutions, manual model building is challenging due to the necessity of using different thresholds for recognizing the map density and the connectivity between nucleotide bases, ribosyl groups, and phosphate backbone^15^. As expected, at a lower threshold display of the cryo-EM density, the sugar-phosphate backbone is visible. At a higher threshold, Watson–Crick base pairs and even individual bases are clearly resolvable (Supplementary Fig. 4 and Supplementary Movie 1). Despite this rich detail, we were not able to successfully build complete models manually or with software previously developed for automatically building RNA coordinates for maps at higher resolution^16,17^. To make the RNA model building easier, faster, and more accurate, we used an RNA-specific modeling tool, auto-DRRAFTER (Supplementary Fig. 5) to build the initial models for apo and SAM-bound SAM-IV riboswitches, followed by optimization with Phenix^18^. The quality of these models was validated using MolProbity^19^ (Supplementary Table 1) and the consistency between the map and model was assessed by cross-correlation coefficients (Supplementary Fig. 6a, b). The Q-score developed for evaluating the density resolvability for the two building elements of each nucleotide (base and backbone) was used to assess the quality of the map and model per nucleotide^20^. The fact that the Q-score across the entire structure of this small RNA (Supplementary Fig. 6c, d) was similar to an expected Q-score of 0.40–0.46 from cryo-EM analyses of the RNAs in ribosomes at comparable resolution further supports the reliability of the structure. Interestingly, we observed that the Q-scores fluctuate every ~15 nt (Supplementary Fig. 6c). Looking at the model (Fig. 2), the low scores seem to coincide with tight turns, loops or kinks where the model may be more dynamic, resulting in lower resolvability. Where the model, on the other hand, has more favorable and stable base pairs, the Q-score is higher.

The SAM-IV riboswitch adopts a double pseudoknot fold involving five helices (P1, P2a, P2b, P3, P5) and two long-range pseudoknot pairings (PK-1, PK-2) (Fig. 2, Supplementary Fig. 7, and Supplementary Movie 1). Starting at the 5’ terminal region of the riboswitch, the ascending strand of P1 with four base pairs is formed. After a turn containing four nucleotides, the riboswitch forms the ascending strand of P2a, followed by one strand of P2b, which is separated by four nucleotides. Following this strand of P2b, C24 to G28 form one strand of the first pseudoknot PK-1. Then the riboswitch continues as the other strand of P2b and descending strand of P2a, followed by the descending strand of P3. After two nucleotides, the riboswitch forms one strand of the other pseudoknot PK-2, after which it continues directly as the ascending strand of P3 and the other strand of PK-1. Then the riboswitch forms the descending strand of P1 and hairpin P5. Finally, at the 3’ end of the riboswitch, the other strand of PK-2 is formed. Notably, the binding of SAM does not disrupt the base pairing and base stacking in SAM-IV; the preservation of the overall fold is visually apparent in the maps and the automated models (Fig. 2, Supplementary Fig. 8, and Supplementary Fig. 9). The phenomenon that no significant conformational change occurs upon ligand binding has also been observed in other riboswitch aptamers, including SAM-I^21^ and lysine riboswitches^22,23^.

This newly resolved SAM-IV tertiary structure offers an informative comparison to SAM-I and SAM-I/IV structures previously solved by X-ray crystallography. Compared with SAM-I^7^, SAM-IV lacks the P4 helix but generates a new pseudoknot PK-2 that is connected to the P5 stem-loop. In addition to this change, the kink-turn motif in SAM-I consists of six nucleotides, while the turn in SAM-IV has only four nucleotides, and has a backbone path unlike the kink in the other structure (Supplementary Fig. 8a, b). In the SAM-I/IV class^10^, the P2 helix is not separated into two helices P2a and P2b; it lacks the PK-1 pseudoknot but has a P4 helix when compared to SAM-IV (Supplementary Fig. 8a, c). Despite these global differences in the topology of the SAM-I, SAM-I/IV, and SAM-IV structures, all three show at their core two parallel co-axial domains (red and orange, Supplementary Fig. 8) positioned to bind the SAM ligand.

### Determination of the ligand location

**Fig. 3:**
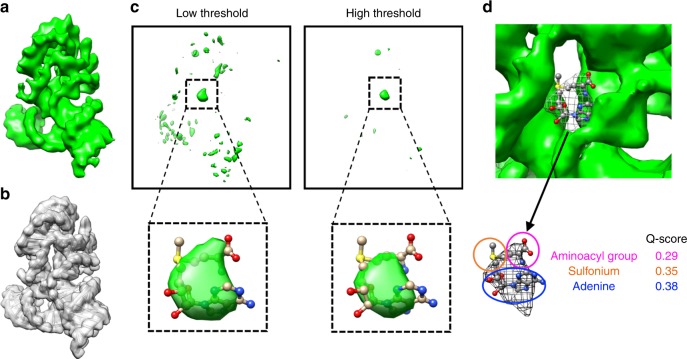
Determination of the ligand location in the ligand-bound SAM-IV cryo-EM map. **a** Ligand-bound SAM-IV cryo-EM map. **b** Map computed from the SAM-IV RNA-only model from **a**. **c** Difference map between **a** and **b** shown at two different thresholds. The insets show the prominent density in the difference map into which are fitted the ligand model derived from SAM-I crystal structure (PDB code: 3GX5). **d** Segmented SAM density from **a** is highlighted in a wire frame representation, fitted with the ligand model derived from SAM-I crystal structure (PDB code: 3GX5). Q-scores of the ligand components between the map and model are calculated.

We sought to determine if the cryo-EM data might allow the identification of the SAM binding pocket, as it would be useful for future studies seeking to discover potential drug binding sites in riboswitches and other small RNA targets. We first modeled the SAM-IV riboswitch RNA coordinates only into the ligand-bound map and then computed a difference map between the model-derived map and the raw density map (Fig. 3a–c), assuming that the differential density is attributable to the ligand and/or portions of the model that exist in multiple conformations (usually surface loops) and/or model inaccuracy. A prominent density was found to be in the central region of the difference map and coincided with the location of the ligand derived from the SAM-I crystal structure (PDB code: 3GX5) (Fig. 3c). To evaluate whether this central density may be attributed to the SAM ligand, the SAM ligand coordinates were converted to a density map at 4-Å resolution. The difference map was exhaustively searched for the best match with the ligand density using Chimera^24^ and Situs^25^. The highest cross-correlation was found at this putative site. A comparison between the apo and SAM-bound maps also reveals the same ligand binding site (Supplementary Fig. 9). In this case, the difference in resolution between the two structures, and slight differences in conformation of the RNA might complicate the interpretation (Supplementary Fig. 9c–f). We further verified the position of the ligand as a separate density peak in an automatically segmented map (Fig. 3d) using the established software, *Segger*^26^. The Q-scores of adenine, sulfonium, and aminoacyl group, three moieties of the ligand model, are 0.38, 0.35, and 0.29, respectively (Fig. 3d), which are comparable to the expected Q-score (0.40) of ribosomal RNA models at 4.1-Å resolution (Supplementary Fig. 6c, d), supporting the reliability of SAM positioning in our cryo-EM map.

Our data support the previous hypothesis that SAM-I, SAM-I/IV, and SAM-IV share a common ligand-binding pocket while adopting distinct peripheral architectures^27^ (Supplementary Fig. 8). The variations of peripheral structures result in alternative ligand recognition mechanisms and potentially different regulatory switch subdomains to regulate the expression of target mRNAs^10^.

## Discussion

In human cells, about 98% of the transcriptional products are non-coding RNAs^28^, and most core molecular biological processes in viruses and living systems also involve non-coding RNAs. Although they are found to be increasingly important in a variety of pathophysiological processes, such as gene regulation, protein homeostasis, and human diseases, and offer a wealth of new targets for small molecule inhibition, the functions of most non-coding RNAs are still unknown^29^. It has been reported that RNAs with known functions appear to have evolutionarily conserved secondary structures^30^. The acquisition of high-resolution structures would facilitate our understanding of RNA structure-function relationships. In this study, we expand the applicability of cryo-EM (in the absence of phase plate) to RNA, with a minimum molecular size of ~40 kDa, which is also smaller than the smallest protein (Streptavidin, 52 kDa) determined by cryo-EM at near-atomic resolution^31^. Notably, the location of the small SAM ligand could also be deduced from our 4.1-Å holo cryo-EM structure. Our results suggest that cryo-EM together with modeling tools can open up a new avenue for routine structure determination of non-coding RNA domains and their potential drug-binding pockets that is no longer limited by the requirements of small molecular weight in nuclear magnetic resonance (NMR) or crystallization in X-ray crystallography.

## Methods

### Sample preparation

RNA was prepared as previously described^13^. Briefly, DNA templates were constructed through PCR assembly using primers listed in the Supplementary Table 2 and then purified with AMPureXP beads (Beckman Coulter). RNA was transcribed with T7 RNA polymerase at 37 °C for 4 h in a buffer containing 0.2 μM DNA template, 40 mM Tris·HCl (pH 8.1), 25 mM MgCl_2_, 3.5 mM spermidine, 0.01% TritonX-100, 40 mM DTT, 4% PEG 8000, 3 mM NTPs, and 5 U/μL T7 RNA polymerase (NEB). RNA was then purified with Zymo RNA Clean and Concentrator columns (Zymo Research) following the manufacturer’s instructions.

### Cryo-EM data collection

Three microliters of the SAM-IV riboswitch RNAs at 40 μM were applied onto glow-discharged 200-mesh R2/1 Quantifoil grids. The grids were blotted for 2–4 s and rapidly cryocooled in liquid ethane using a Vitrobot Mark IV (Thermo Fisher Scientific) at room temperature and ~100% humidity. The samples were screened using a Talos Arctica cryo-electron microscope (Thermo Fisher Scientific) operated at 200 kV. To test the feasibility of cryo-EM for determining RNA structures, the apo SAM-IV riboswitch was first imaged in a Talos Arctica cryo-electron microscope with and without phase plate, respectively. For the high-resolution study, both of the apo and SAM-bound SAM-IV riboswitches were imaged in a Titan Krios cryo-electron microscope (Thermo Fisher Scientific) with GIF energy filter (Gatan) at a magnification of ×130,000 (corresponding to a calibrated sampling of 1.06 Å per pixel). Micrographs were recorded by EPU software (Thermo Fisher Scientific) with a Gatan K2 Summit direct electron detector, where each image was composed of 30 individual frames with an exposure time of 6 s and a dose rate of 7.6 electrons per second per Å^2^. Finally, a total of 7200 movie stacks for the apo state and 6030 movie stacks for the bound state were collected with a defocus range of −1.5 to −3.5 μm.

### Image processing

All micrographs were motion-corrected using MotionCor2^32^ and the contrast transfer function (CTF) was determined using CTFFIND4^33^. All particles were autopicked using the NeuralNet option in EMAN2^34^ and further checked manually. The resulting number of boxed particles were 2,102,569 for the apo state and 1,830,706 for the SAM-bound state. Then, particle coordinates were imported to Relion^35^, where three rounds of 2D classification were performed to remove 2D class averages with less resolved features. Using 1,137,602 particles of the apo state, we first generated an ab-initio map using cryoSPARC^36^ and refined it with Relion, yielding a 3.9-Å map. Then starting with the same ab-initio map, we reprocessed the same set of particles using cryoSPARC and yielded a final map with 3.7-Å resolution from 796,923 particles. For the SAM-bound state, we selected 984,150 particles by Relion and processed them using cryoSPARC to yield a 4.1-Å map from 588,580 particle images. Both map determinations used the 3D non-uniform refinement and local refinement options of cryoSPARC. The cited resolutions for the final maps were estimated by the 0.143 criterion of FSC curve in cryoSPARC. The 3.7-Å and 4.1-Å low-pass filters were applied, respectively, to the final 3D maps for better display (See more information in Supplementary Fig. 2 and Supplementary Table 1).

### Model building

The initial SAM-IV riboswitch models were built with auto-DRRAFTER^13^. We built both fully automated models and models using information from the previously determined crystal structure of the homologous SAM-I riboswitch (PDB code: 3GX5) for the apo and SAM-bound states. For the fully automated models, 2000 models were built per round, and a total of three and four rounds of modeling were performed for the apo and SAM-bound states, respectively. For the models built with information from the previously solved SAM-I structure, P5 was first approximately fit into the density maps for the apo and SAM-bound states. We modeled the conformation of the SAM binding pocket by taking all residues that were within 5 Å of SAM in the SAM-I crystal structure (residues 6–8, 11–12, 44–47, 57–59, 87 in SAM-I numbering, corresponding to residues 2–4, 7–8, 63–65, 77 in SAM-IV numbering) and mutating the nucleotides to the SAM-IV sequence. These nucleotides were kept fixed as a rigid body in the first round of modeling for the SAM-IV apo state, and in all but the final round of modeling for the SAM-bound state. Three rounds of modeling were performed for the apo and SAM-bound states, with 2000 models built per round. These models agree closely with the fully automated models. All final models were built into half maps to prevent overfitting (Fig. S5).

Next, the top-scoring models of the apo and SAM-bound states built with information from the SAM-I crystal structure were selected for further refinement by ERRASER and Phenix^37^ to optimize the geometry while improving the fit to the cryo-EM density. As the current resolution was not sufficient to determine the conformation of the SAM binding pocket, residues 2–4, 7–8, 63–65, 77 and SAM derived from SAM-I crystal structure were fixed during the optimizations. The final models were evaluated by MolProbity and Q-score^20^. All figures were prepared using PyMol^38^ or Chimera^24^. Secondary structure diagrams were prepared with RiboDraw [https://github.com/ribokit/RiboDraw].

### Reporting summary

Further information on research design is available in the Nature Research Reporting Summary linked to this article.

## Supplementary information

Supplementary Information

Peer Review

Reporting Summary

Description of Additional Supplementary Files

Supplementary Movie 1

## Data Availability

The data supporting the findings of this manuscript are available from the corresponding authors upon reasonable request. Cryo-EM structures and atomic models have been deposited to the Electron Microscopy Data Bank under accession codes EMD-20755, EMD-20756, and the Protein Data Bank under accession codes 6UES, 6UET, respectively.
