# Supplementary material for: Cryo-EM structure of a 40 kDa SAM-IV riboswitch RNA at 3.7 Å resolution
Source: Nat Commun. 2019 Dec 3;10:5511. doi: 10.1038/s41467-019-13494-7 (PMC6890682; doi:10.1038/s41467-019-13494-7)
Supplement: Supplementary file 1 — Supplementary Information [file 41467_2019_13494_MOESM1_ESM.pdf]

Supplementary Information

Cryo-EM Structure of a 40-kDa SAM-IV Riboswitch RNA at 3.7 Å Resolution

Zhang *et al.*

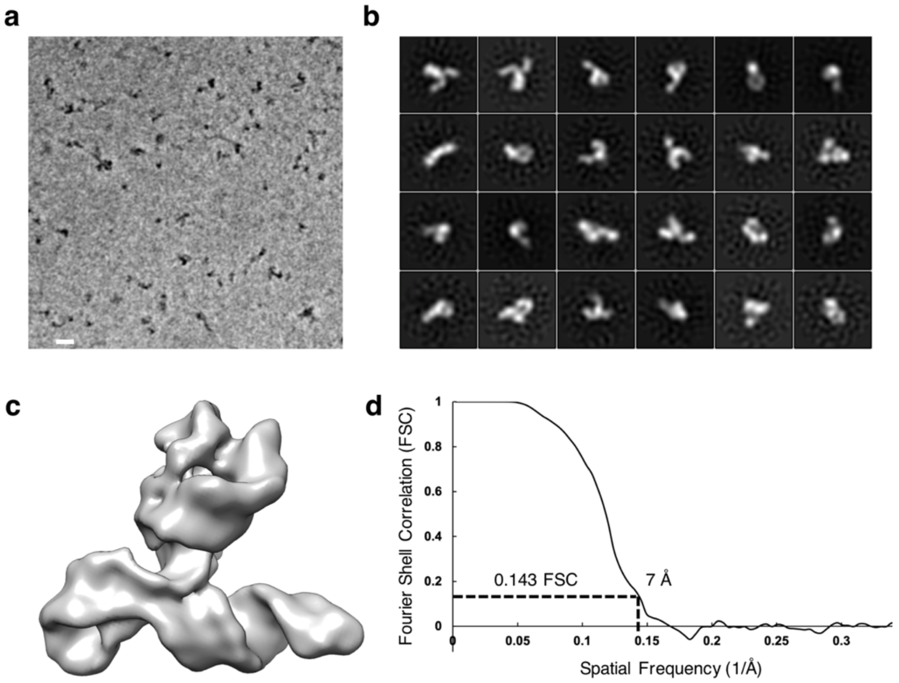

Supplementary Figure 1. Single-particle cryo-EM analysis of the apo SAM-IV riboswitch collected on Talos Arctica with Volta phase plate. a Representative motion-corrected cryo-EM micrograph. Scale bar represents 100 Å. **b** Reference-free 2D class averages computed in Relion. **c** Final 3D reconstruction. **d** Gold standard FSC plot for the 3D reconstruction calculated in cryoSPARC.

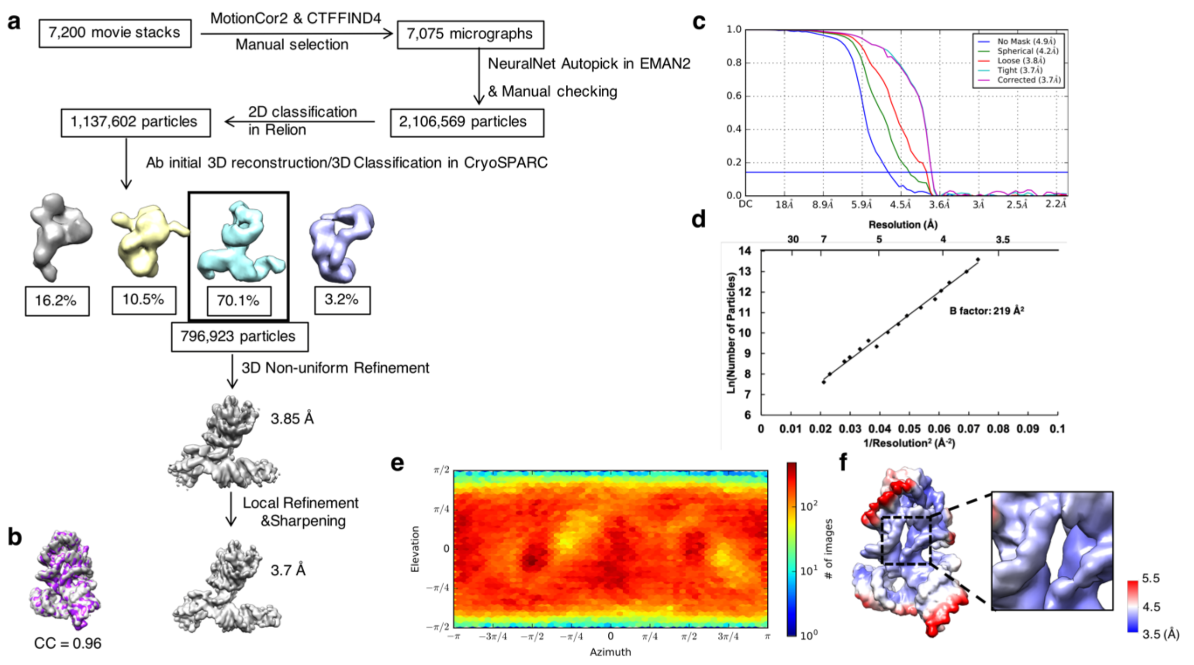

Supplementary Figure 2. Single-particle cryo-EM analysis of the apo SAM-IV riboswitch collected on Titan Krios. a Workflow of cryo-EM data processing. **b** Comparison of reconstruction results from Relion 3.9-Å map (purple) and CryoSPARC 3.7-Å map (gray). **c** Gold standard FSC plots calculated in CryoSPARC. **d** Plot of the particle number vs the reciprocal squared resolution. The B-factor was calculated as 2 X the linear fitting slope. **e** Euler angle distribution of the particle images. **f** Resolution map for the 3D reconstruction, with an expanded view of the central portion.

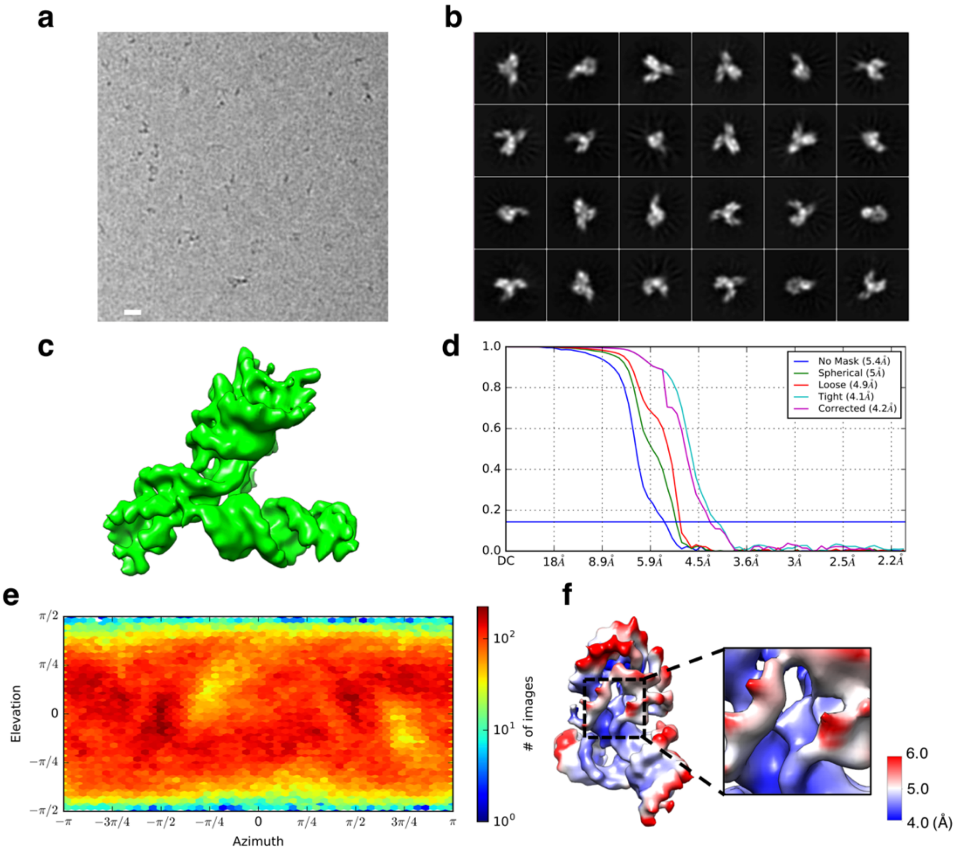

Supplementary Figure 3. Single-particle cryo-EM analysis of the SAM-bound SAM-IV riboswitch collected on Titan Krios. a Representative motion-corrected cryo-EM micrograph. Scale bar represents 100 Å. **b** Reference-free 2D class averages computed in Relion. **c** Final 3D reconstruction. **d** Gold standard FSC plots calculated in CryoSPARC. **e** Euler angle distribution of the particle images. **f** Resolution map for the 3D reconstruction, with an expanded view of the central ligand-binding pocket.

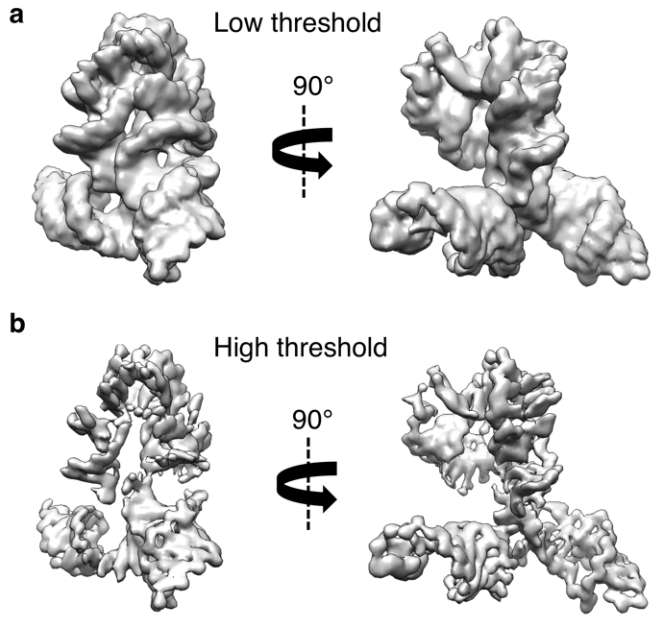

Supplementary Figure 4. Visualization of cryo-EM map of the apo SAM-IV riboswitch at a low threshold (a) and a high threshold (b).

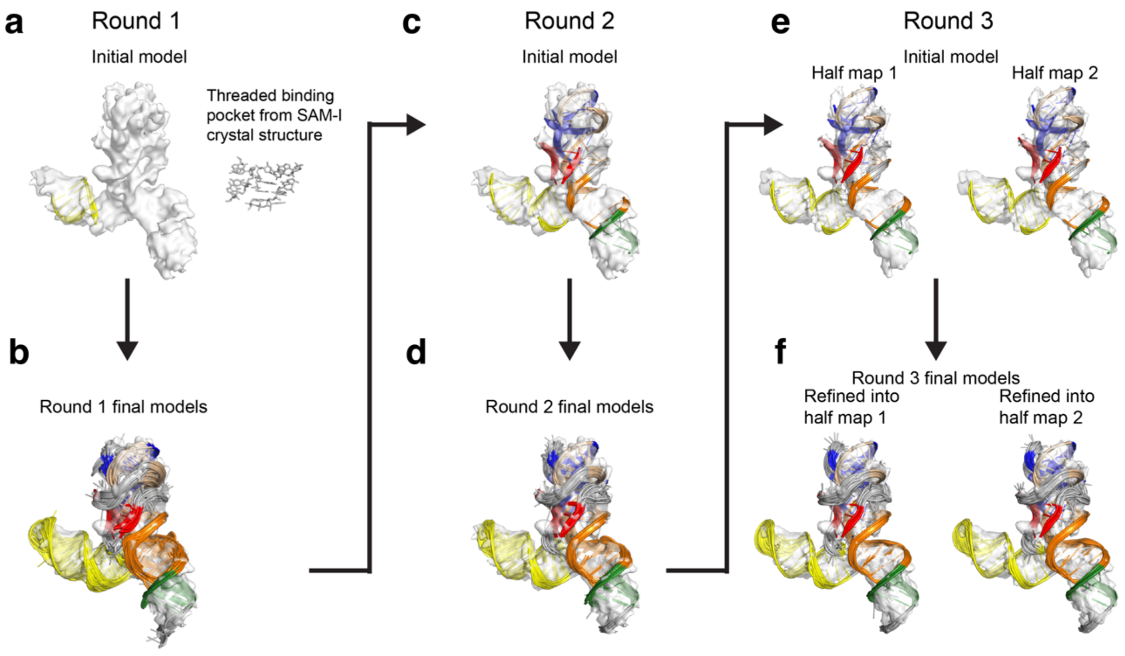

Supplementary Figure 5. Schematic of auto-DRRAFTER model building for the SAM-IV riboswitch. **a** A single helix (P5) was placed into the density map either manually or automatically (for fully automatic models). For models built using information from a homologous SAM-I structure, the threaded binding pocket was also input as a rigid body. **b** Auto-DRRAFTER automatically filled in missing RNA coordinates. A total of 2,000 models were built in each round. The top ten scoring models for SAM-IV built using the threaded SAM-I binding pocket and manually placed initial helix are shown here. **c** Regions that converged in the first round of modeling were automatically extracted and used as the initial structure for the second round of modeling. **d** Ten best scoring models from round 2. **e** Regions that converged in the second round of modeling were automatically extracted and used as the initial structure for the third and, in this case, final round of modeling. **f** The final models were refined into half maps to prevent overfitting.

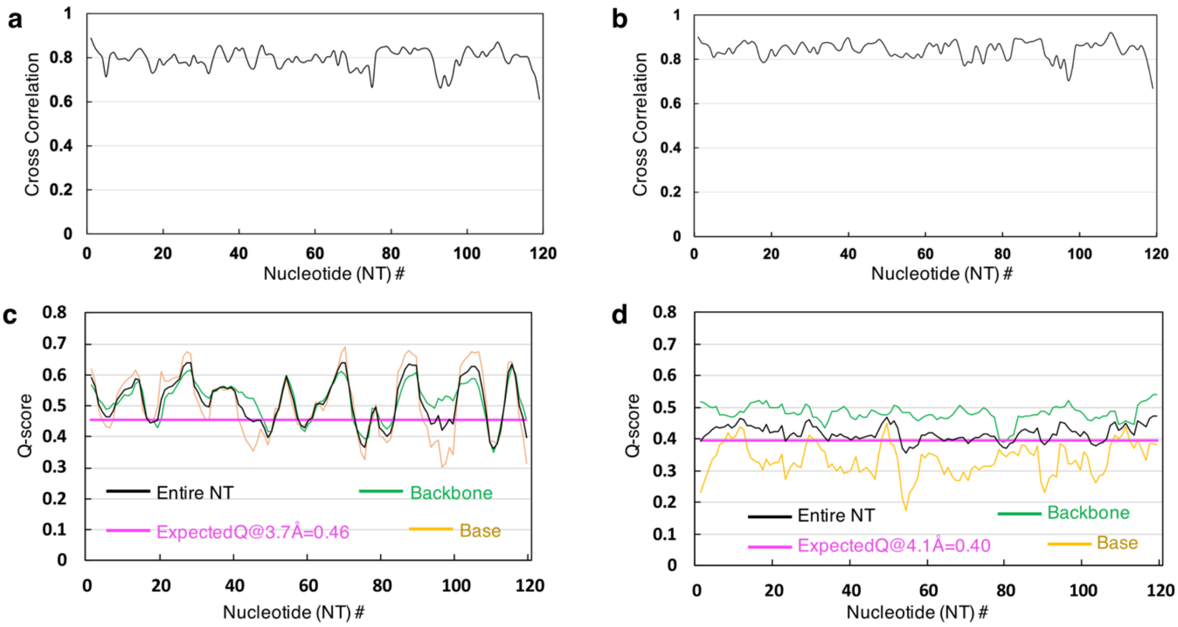

Supplementary Figure 6. Model validation of the apo and SAM-bound SAM-IV riboswitches. a Per-nucleotide cross-correlation coefficient between the model and 3.7-Å map of apo SAM-IV riboswitch. **b** Per-nucleotide cross-correlation coefficient between the model and 4.1-Å map of SAM-bound SAM-IV riboswitch. **c** Q-score for each nucleotide in model and 3.7-Å map of apo SAM-IV riboswitch; the pink line represents the expected Q-score at 3.7-Å resolution (0.46) based on the correlation between Q-scores and map resolution. **d** Q-score for each nucleotide in model and 4.1-Å map of SAM-bound SAM-IV riboswitch; the pink line represents the expected Q-score for models at 4.1-Å resolution (0.40).

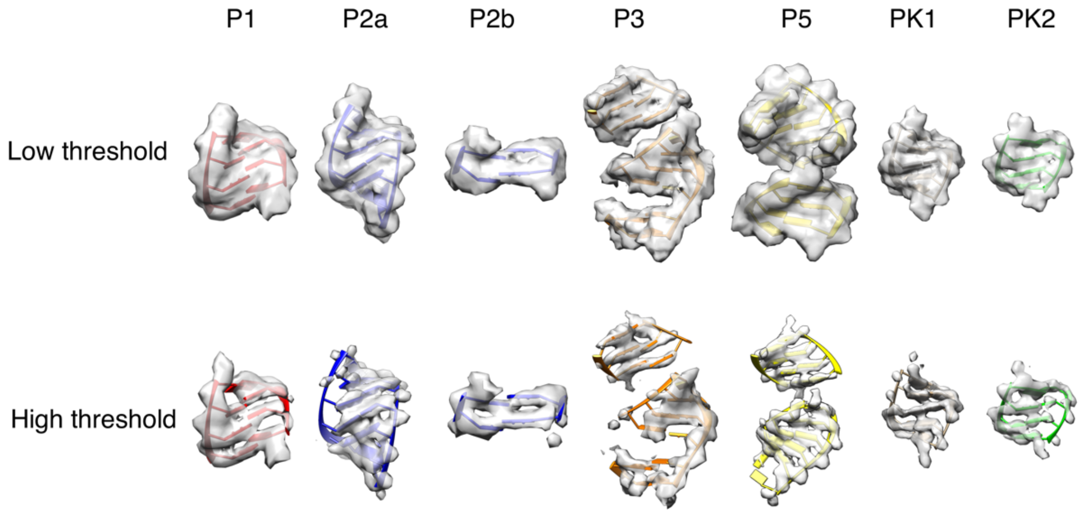

Supplementary Figure 7. Representative density regions of the apo SAM-IV riboswitch at two different thresholds.

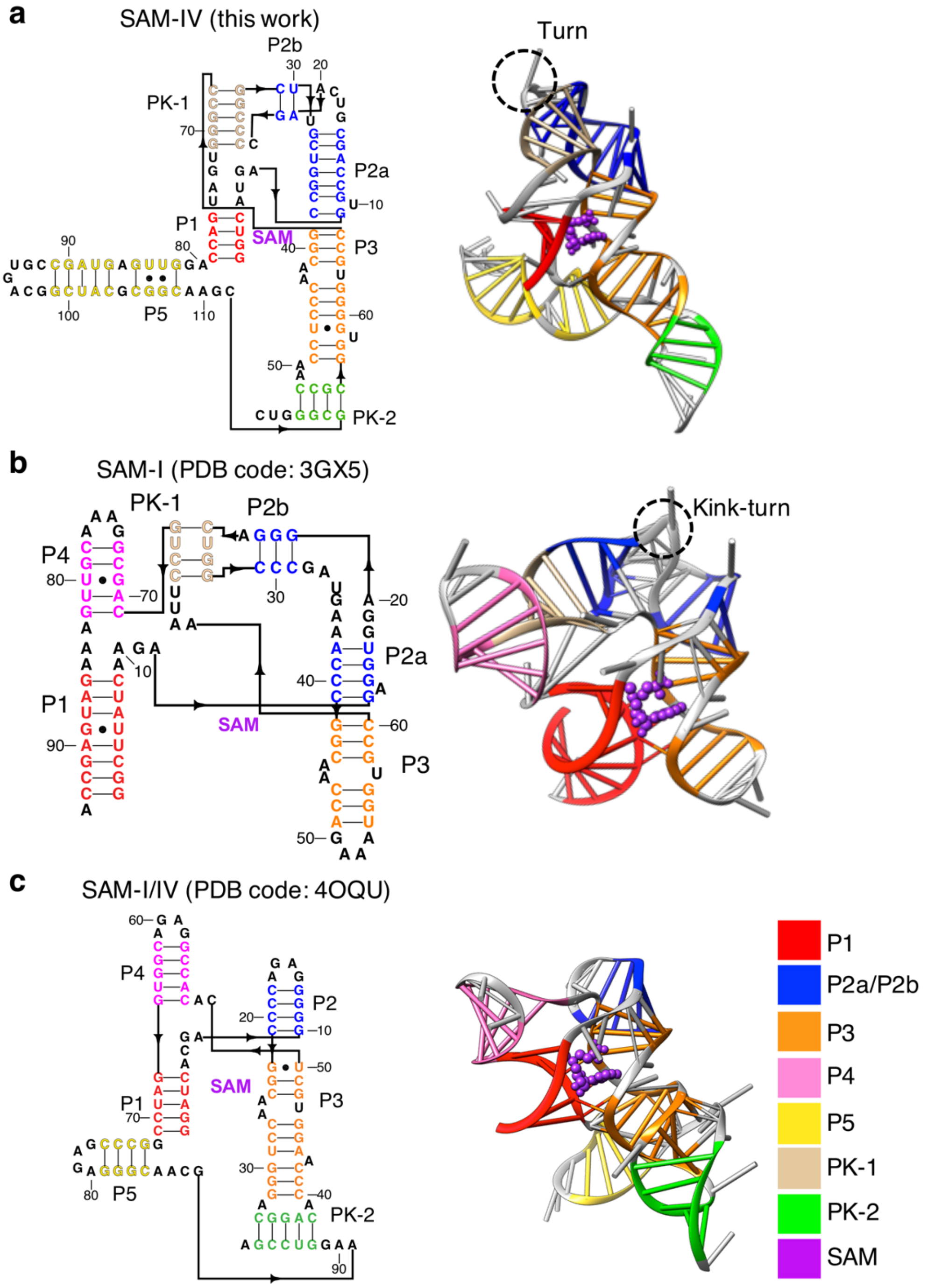

Supplementary Figure 8. Comparison of the secondary and tertiary structures of the SAM-bound SAM-IV (a) with SAM-I (b) and SAM-I/IV (c). Left panel, the secondary structures with the domains colored differently. The black arrow indicates the direction of the backbone. Right panel, the models colored by the same scheme as in the left panel. The SAM ligand is shown as ball in purple color. P, paired; PK, pseudoknot.

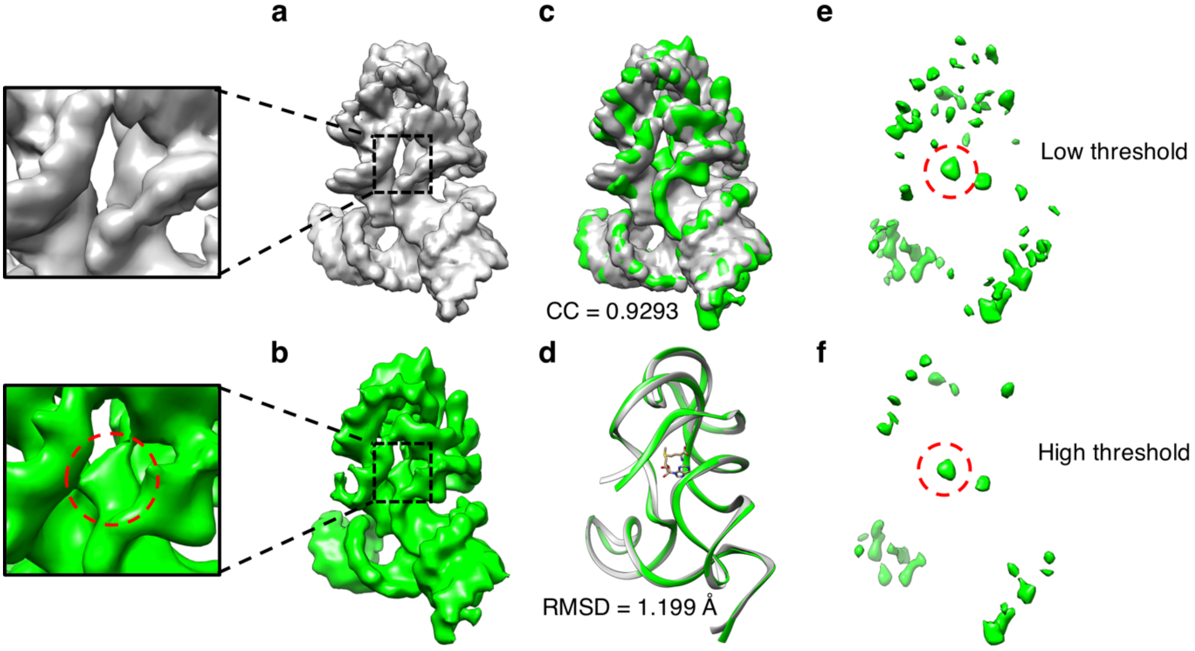

Supplementary Figure 9. Comparison of the cryo-EM maps between the apo and SAM-bound SAM-IV riboswitches. a Overall cryo-EM map of the apo state with a zoom-in view of the central portion. **b** Overall cryo-EM map of the SAM-bound state with a zoom-in view of the central putative SAM-binding region (red circle). **c** Comparison of the cryo-EM maps between the apo state and SAM-bound state, with the cross-correlation coefficient listed. **d** Comparison of the models between the apo state and SAM-bound state, with the RMSD listed. **e-f** Difference map between the apo state and SAM-bound state at two different thresholds, highlighting the central putative SAM-binding region.

Supplementary Table 1. Cryo-EM data collection, processing, and model validation

	Apo SAM-IV riboswitch	SAM-bound SAM-IV riboswitch
**Data collection and processing**		
Microscope	Titan Krios	Titan Krios
Voltage (kV)	300	300
Camera	Gatan K2 Summit	Gatan K2 Summit
Pixel size (Å)	1.06	1.06
Total Dose (e-/Å^2)	45.6	45.6
Defocus range (µm)	-1.5 - -3.5	-1.5 - -3.5
Number of micrographs	7,200	6,030
Number of initial particles	2,102,569	1,830,706
Symmetry	C1	C1
Number of final particles	796,923	588,580
Resolution (0.143 FSC, Å)	3.7	4.1
**Atomic model refinement**		
Software	phenix.real_space_refine	phenix.real_space_refine
Clash score	6.25	9.82
MolProbity score	2.49	2.66
Probably wrong sugar puckers (%)	2	5
Bad bonds (%)	0	0
Bad angles (%)	0	3

Supplementary Table 2. Sequences for DNA template, RNA, and PCR assembly primers of SAM-IV riboswitch

Description	Sequence
SAM-IV riboswitch DNA template, full sequence	TTCTAATACGACTCACTATAGGTCATGAGTGCCAG CGTCAAGCCCCGGCTTGCTGGCCGGCAACCCTCCA ACCGCGGTGGGGTGCCCCGGGTGATGACCAGGTTG AGTAGCCGTGACGGCTACGCGGCAAGCGCGGGTC
SAM-IV riboswitch RNA, full sequence	GGUCAUGAGUGCCAGCGUCAAGCCCCGGCUUGCU GGCCGGCAACCCUCCAACCGCGGUGGGGUGCCCC GGGUGAUGACCAGGUUGAGUAGCCGUGACGGCUA CGCGGCAAGCGCGGGUC
DNA template assembly primer 1	TTCTAATACGACTCACTATAGGTCATGAGTG
DNA template assembly primer 2	GAGGGTTGCCGGCCAGCAAGCCGGGGCTTGACGCT GGCACTCATGACCTATAGTGAGTCG
DNA template assembly primer 3	GCCGGCAACCCTCCAACCGCGGTGGGGTGCCCCGG GTGATGACCAGGTTGAGTAGCCGTG
DNA template assembly primer 4	GACCCGCGCTTGCCGCGTAGCCGTCACGGCTACTC AACCTGGTCATC
