# Supplementary material for: Cryo-EM structure of a 40 kDa SAM-IV riboswitch RNA at 3.7 Å resolution
Source: Nat Commun. 2019 Dec 3;10:5511. doi: 10.1038/s41467-019-13494-7 (PMC6890682; doi:10.1038/s41467-019-13494-7)
Supplement: Supplementary file 4 — Description of Additional Supplementary Files [file 41467_2019_13494_MOESM4_ESM.docx]

**Description of Additional Supplementary Files**

File name: Supplementary Movie 1
Description: 3.7-Å cryo-EM map and model of 40-kDa SAM-IV riboswitch RNA. Different regions of the map are shown with varying contour levels along with the model to illustrate the high fidelity of the match between model and map.
