# Supplementary material for: Cryo-EM structure of a 40 kDa SAM-IV riboswitch RNA at 3.7 Å resolution
Source: Nat Commun. 2019 Dec 3;10:5511. doi: 10.1038/s41467-019-13494-7 (PMC6890682; doi:10.1038/s41467-019-13494-7)
Supplement: Supplementary file 2 — Peer Review [file 41467_2019_13494_MOESM2_ESM.pdf]

Reviewers' comments:

Reviewer #1 (Remarks to the Author):

The work by Zhang et al. presents a newly determined structure of a riboswitch regulating sulfur metabolism in bacteria, with potential antibiotic target applications. Furthermore, the authors demonstrate cutting-edge technical performance in cryo-electron microscopy that breaks the current molecular weight paradigm and will encourage other researchers to try similar applications. The results are of high-quality and are clearly presented. I am confident that the paper will be of great interest to researchers from multiple fields and therefore recommend its publication in Nature Communications with a few minor revisions.

Minor points:
1. Line 99: "After a turn containing six nucleotides, …" The figure shows the turn containing four nucleotides. Please correct the text.
2. Lines 107-108 "the preservation of the overall fold is visually apparent in the maps and the automated models (Fig. 2 and Extended Data Fig. 8a)." Neither Fig.2 nor Ext. Data Fig. 8 show a comparison between the apo and SAM-bound models. Please either add a panel to Fig. 2 or another Extended Data Figure with the apo and SAM-bound models overlaid to clearly illustrate the structural differences or their absence.

Reviewed by Radostin Danev

Reviewer #2 (Remarks to the Author):

In this manuscript, Zhang et al. used single particle cryo-EM to reveal the structures of a 40-kDa RNA-only SAM-IV riboswitch in different ligand binding states. In these resolved structures, the resolution is high enough to identify/trace the sugar phosphate backbone and some base pairs of RNA. The authors combined various methods to build and validate the atomic models from the cryo-EM results. The information was integrated to further identify the SAM-binding pocket. This work provides important insight to both the mechanism of riboswitch ligand recognition and demonstrates the power of single particle cryo-EM in determining RNA structures in small size. Overall, the manuscript is well-written and presented clearly.

I have a few remarks for the authors to address:

Major remark is in the section that describes the identification of ligand binding pocket (from line 121). Without pre-knowledge, the direct ways to locate the binding pocket are: 1) a high enough resolution map shows separated density(s) that define a clear feature of the ligand; 2) compare the apo-/ligand-bound- state structures in the same condition to find significant density(s) corresponding to the ligand. I agree with the authors' statement that the difference in resolution and slight differences in conformations might influence the analysis. However, the difference between apo and ligand-bound map is the "real" data from the cryo-EM reconstruction, while the model-derived map is a deduced one. The authors stated that (line 129) "A comparison between the apo and SAM-bound maps also reveals the same ligand binding site", but they didn't show the difference map between the apo and SAM-bound maps. Such a difference map should be provided in the supplementary information.

I'm curious if the SAM-I crystal structure is unknown, how much confidence the SAM could be locate with the 4.1 angstrom map. In Fig. 3c, at the threshold of 1.5, there are two relatively large densities but both smaller than the size of SAM. Besides, the separated ligand density in Fig. 3c and 3d seems to have different shapes, can the author make an explanation for this? I think the statements

“unbiased identification of the SAM binding pocket” and “Notably, the location of the ~ 0.4-kDa ligand could also be determined in our 4.1-Å holo cryo-EM map” should be tuned down.

Minor remarks:

1) In Ext. Fig. 6c, the Q score distribution along NT# seems to have a fluctuation in every ~15 NT. Is there an explanation about it? I’m also curious about whether the Q score distribution is related to the local resolution distribution.

2) As this work is a record-making cryo-EM study of small RNA molecules, the detailed validation results of the maps (i.e. Euler angle distribution, un-masked FSC) should be provided in the supplemental materials for others to understand better of the dataset.

3) The claimed defocus ranges for data collection in Methods section (line 177-178) are different from the ranges provide in Table S1.

Hong-Wei Wang

Reviewer #3 (Remarks to the Author):

Single-particle cryo-electron microscopy (cryo-EM) has solved numerous near-atomic or atomic resolution structures of biological molecules recently. Most of the structures are focused on protein or protein-RNA complex. Using cryo-EM to solve the structure of solo RNA molecules, especially small RNA molecules with molecular weight less than 50 KDa will promote the development of the entire RNA research field. The manuscript by Zhang, K. et al., titled "Cryo-EM Structure of a 40-kDa SAM-IV Riboswitch RNA at 3.7 Å Resolution" reports the structures of apo and SAM-bound SAM-IV riboswitches (119 nt, approximately 40 KDa) to 3.7 Å and 4.1 Å resolution with cryo-EM method respectively. Besides, the comparison was made between the structures of SAM-I, SAM-I/IV and SAM-IV riboswitches. SAM-IV adopted similar ligand-binding core but different peripheral tertiary contacts with SAM-I and SAM-I/IV riboswitch. Based on these results, the authors proposed the feasibility of solving the structure of small RNA molecules with cryo-EM, which may facilitate the structure-based drug design for RNA.

It was known that the SAM-I, SAM-VI and SAM-I/IV belong to the same superfamily of SAM riboswitch and previous research shown they share some common structural features [Weinberg, Z. et al. RNA 14, 822-828 (2008), Trausch, J. J. et al. PNAS 111, 6624-6629(2014) and Price, I. R. et al. Biochimica et biophysica acta 1839, 931-938 (2014)]. Both the structures of SAM-I (PDB: 3GX5) and SAM-I/IV (PDB: 4OQU) riboswitches were solved by X-ray crystallography method already. The above information would definitely assist the structural modeling of SAM-IV riboswitch in this manuscript. How about the RNA molecules that have no sequence similarity with other structure-solved RNA molecules? Is it possible to apply this method to a novel RNA molecule and get the same resolution result? The second question is that it’s obvious that the current resolution in the manuscript is not high enough to provide the exact structural detail for the structure-based drug design of RNA, please explain how does it assist the drug design of RNA in the current stage.

Major Comments/Suggestions:

1. What’s the local resolution of the ligand-binding pocket in the apo and SAM-bound structure? Maybe it’s better to show an expanded view of it.

2. In the discussion, it was suggested to relate the current cryo-EM structural results of SAM-IV riboswitch with the previous functional study such as the results in the reference [Weinberg, Z. et al.

RNA 14, 822-828 (2008)] if the authors are not planning to start other validation experiments.

3. In Extended Data Figure 2a, what's the difference between the refinement result of Relion and CryoSparc? How to improve the resolution from 3.93 Å to 3.7 Å? Is it possible to align them together and give a comparison?

4. In the panel d of Extended Data Figure 3, it seems the peripheral resolution is lower than 5 Å. But in Page 6 line 115, it was mentioned that "the kink-turn motif between P2a and P2b helices in the SAM-I is substituted for a distinct turn in SAM-IV". How did you define the "distinct turn"?

5. In Extended Data Figure 6c, it seems the Mean.Q (3.7 Å)=0.46 curve is below the average position of the Entire NT, Backbone and Base curves. Please check them.

Minor Comments/Suggestions:

1. Page 2, line 38, change "possible" to "potential".
2. Since there is enough space in the Extended Data Figure 8, the topology figure and the tertiary figure of three riboswitches in each panel can be shown side to side to make the view larger.
3. In stem P5, do A86 and G104 form the non-canonical base pair? How about the terminal bases G81 and A109?
4. The Abstract in Nature Communications was requested not to contain references, please check the format of the abstract.
5. Page 7, line 155, change "and" to "or".
6. Page 7, line 158, "RNA was prepared as describe in 13". It seems the authors missed the word "reference".

Responses to the reviewers' comments on Manuscript entitled "Cryo-EM Structure of a 40-kDa SAM-IV Riboswitch RNA at 3.7 Å Resolution"

Reviewer #1 (Remarks to the Author):

The work by Zhang et al. presents a newly determined structure of a riboswitch regulating sulfur metabolism in bacteria, with potential antibiotic target applications. Furthermore, the authors demonstrate cutting-edge technical performance in cryo-electron microscopy that breaks the current molecular weight paradigm and will encourage other researchers to try similar applications. The results are of high-quality and are clearly presented. I am confident that the paper will be of great interest to researchers from multiple fields and therefore recommend its publication in Nature Communications with a few minor revisions.

Minor points:

1. Line 99: "After a turn containing six nucleotides, …" The figure shows the turn containing four nucleotides. Please correct the text.

Response: Thank you, it is 4 nucleotides. This is changed on page 5.

2. Lines 107-108 "the preservation of the overall fold is visually apparent in the maps and the automated models (Fig. 2 and Supplementary Figure 8a)." Neither Fig.2 nor Ext. Data Fig. 8 show a comparison between the apo and SAM-bound models. Please either add a panel to Fig. 2 or another Supplementary Figure with the apo and SAM-bound models overlaid to clearly illustrate the structural differences or their absence.

Response: We thank this reviewer for pointing out this omission. We have added panel 9d to Supplementary Figure 9, comparing the two models and listing the RMSD of the two models.

Reviewed by Radostin Danev

Reviewer #2 (Remarks to the Author):

In this manuscript, Zhang et al. used single particle cryo-EM to reveal the structures of a 40-kDa RNA-only SAM-IV riboswitch in different ligand binding states. In these resolved structures, the resolution is high enough to identify/trace the sugar phosphate backbone and some base pairs of RNA. The authors combined various methods to build and validate the atomic models from the cryo-EM results. The information was integrated to further identify the SAM-binding pocket. This work provides important insight to both the mechanism of riboswitch ligand recognition and demonstrates the power of single particle cryo-EM in determining RNA structures in small size. Overall, the manuscript is well-written and presented clearly.

I have a few remarks for the authors to address:

Major remark is in the section that describes the identification of ligand binding pocket (from line 121). Without pre-knowledge, the direct ways to locate the binding pocket are: 1) a high enough resolution map shows separated density(s) that define a clear feature of the ligand; 2) compare the apo-/ligand-bound- state structures in the same condition to find significant density(s) corresponding to the ligand. I agree with the authors' statement that the difference in resolution and slight differences in conformations might influence the analysis. However, the difference between apo and ligand-bound map is the "real" data from the cryo-EM reconstruction, while the model-derived map is a deduced one. The authors stated that (line 129) "A comparison between the apo and SAM-bound maps also reveals the same ligand binding site", but they didn't show the difference map between the apo and SAM-bound maps. Such a difference map should be provided in the supplementary information.
Response: We agree with this reviewer's suggestion. A second difference map, between the apo and SAM-bound cryo-EM maps, is now added in the Supplementary Figure 9e, f. A superimposed map of these two states is also added in Supplementary Fig 9c, showing similar extra density corresponding to the ligand. It should be noted that this difference map also shows other peaks due to the difference in resolution, small changes in the RNA itself, and noise, between the two maps, which are not present in the map minus model difference map in Fig. 3.

The SAM ligand was found to be in the central region of the difference map and coincided with the location of the ligand derived from the SAM-I crystal structure (Fig. 3c). We also further verified the ligand by segmenting the cryo-EM map using *Segger* (Pintilie, G. et al. *J. Struct. Biol.* **170**, 427–438 (2010)), producing a separable segment similar to the density observed in the difference map at the same location (Fig. 3d).

I'm curious if the SAM-I crystal structure is unknown, how much confidence the SAM could be locate with the 4.1 angstrom map. In Fig. 3c, at the threshold of 1.5, there are two relatively large densities but both smaller than the size of SAM. Besides, the separated ligand density in Fig. 3c and 3d seems to have different shapes, can the author make an explanation for this? I think the statements "unbiased identification of the SAM binding pocket" and "Notably, the location of the ~ 0.4-kDa ligand could also be determined in our 4.1-Å holo cryo-EM map" should be tuned down.
Response: We thank the reviewer for this question and thus have undertaken the additional analysis. We found the ligand locations by the difference maps without using the ligand crystal structure (Fig. 3 and Supplement Figure 9). In order to validate whether this central density is indeed attributable to the SAM ligand, we used the ligand crystal structure. To do so, we converted the SAM ligand coordinates to a 4-Å density map, and this density was scanned over the entire difference map using Chimera and Situs (Wriggers et al *J Struct Biol* **125**(2-3):185-195). The highest cross-correlation was found at this putative site. Furthermore, the Q-score

(Pintilie, G.. doi:10.1101/722991) of the ligand as placed, 0.35, is close to the expected Q-score in a 4-Å map, 0.40. (In contrast, the Q-score of the same relative position for the ligand in the apo map is -0.24). Our workflow suggests that we can reveal the location of the ligand without prior ligand crystal information. Of course, we cannot characterize the atomic details of the ligand and its interactions with the RNA because of the limited resolution.

We can segment a similar density from the actual cryo-EM SAM-bound map, as shown in Figure 3d. The size and shape of the density corresponding to the ligand depends on the choice of threshold (A new panel with low threshold has been added in Fig. 3c) and the resolution of the map. We think the small difference in shape between the segmented density (Fig. 3d) and the difference map (Fig. 3c) is due to the segmentation accuracy at this resolution. We have revised the manuscript and described appropriately our findings and interpretations concerning the localization of the ligand on pages 7-8.

Minor remarks:

1) In Ext. Fig. 6c, the Q score distribution along NT# seems to have a fluctuation in every ~15 NT. Is there an explanation about it? I'm also curious about whether the Q score distribution is related to the local resolution distribution.
Response: It is true that the Q-core can be related to the local resolution variation. We have discussed the relationship of Q-score in reference to the map resolution in our manuscript submitted to BioRxiv (Pintilie, G.. doi:10.1101/722991). This reviewer raised a very interesting observation that the Q-scores fluctuate every ~15 NT. Looking at the model more closely, the low points seem to coincide with turns, loops or kinks where the model may be more stressed or dynamic, resulting in lower resolvability. Where the model, on the other hand, indicates more favorable and stable base pairs, the Q-score is higher. We thank the reviewer for the question and we mention this phenomenon on page 5 in the revised text.

2) As this work is a record-making cryo-EM study of small RNA molecules, the detailed validation results of the maps (i.e. Euler angle distribution, un-masked FSC) should be provided in the supplemental materials for others to understand better of the dataset.
Response: We have included the Euler angle distribution and FSC curves derived from the cryoSPARC refinement in the Supplementary Figure 2 and Supplementary Figure 3.

3) The claimed defocus ranges for data collection in Methods section (line 177-178) are different from the ranges provide in Table S1.
Response: We apologize for this mistake. We have corrected the defocus range in Table S1.

Hong-Wei Wang

Reviewer #3 (Remarks to the Author):

Single-particle cryo-electron microscopy (cryo-EM) has solved numerous near-atomic or atomic resolution structures of biological molecules recently. Most of the structures are focused on protein or protein-RNA complex. Using cryo-EM to solve the structure of solo RNA molecules, especially small RNA molecules with molecular weight less than 50 KDa will promote the development of the entire RNA research field. The manuscript by Zhang, K. et al., titled "Cryo-EM Structure of a 40-kDa SAM-IV Riboswitch RNA at 3.7 Å Resolution" reports the structures of apo and SAM-bound SAM-IV riboswitches (119 nt, approximately 40 KDa) to 3.7 Å and 4.1 Å resolution with cryo-EM method respectively. Besides, the comparison was made between the structures of SAM-I, SAM-I/IV and SAM-IV riboswitches. SAM-IV adopted similar ligand-binding core but different peripheral tertiary contacts with SAM-I and SAM-I/IV riboswitch. Based on these results, the authors proposed the feasibility of solving the
structure of small RNA molecules with cryo-EM, which may facilitate the structure-based drug design for RNA.

It was known that the SAM-I, SAM-VI and SAM-I/IV belong to the same superfamily of SAM riboswitch and previous research shown they share some common structural features [Weinberg, Z. et al. RNA 14, 822-828 (2008), Trausch, J. J. et al. PNAS 111, 6624-6629(2014) and Price, I. R. et al. Biochimica et biophysica acta 1839, 931-938 (2014)]. Both the structures of SAM-I (PDB: 3GX5) and SAM-I/IV (PDB: 4OQU) riboswitches were solved by X-ray crystallography method already. The above information would definitely assist the structural modeling of SAM-IV riboswitch in this manuscript. How about the RNA molecules that have no sequence similarity with other structure-solved RNA molecules? Is it possible to apply this method to a novel RNA molecule and get the same resolution result?
Response: Our map was computed without using prior information from these structures. We judged the quality of the maps using the best practices in cryo-EM in terms of FSC from two independent half-maps and the recently-introduced Q-score. We agree that the existence of crystal structures of other members of SAM riboswitches assisted us to build the models with high level of confidence. Our models are validated by the PDB validation test and are within the acceptable range of model accuracy in terms of stereochemistry.

This reviewer raised a very good question: if there is a completely unknown structure, can we derive an RNA model with confidence? We are pleased to report that it is possible to use cryo-EM maps at 3-10 Å resolution to assist in building RNA atomic models with an estimate of their model accuracy. We have carried out some other studies of RNA molecules ranging from 62-388

nucleotides to demonstrate this integrative approach. These results are documented in two separate manuscripts (doi:https://doi.org/10.1101/717801), which are under review.

The second question is that it's obvious that the current resolution in the manuscript is not high enough to provide the exact structural detail for the structure-based drug design of RNA, please explain how does it assist the drug design of RNA in the current stage.
Response: There are multiple steps in the drug discovery process. Once a drug candidate is identified by its medicinal assay, one would like to know where the drug binds and what are the chemical details of the drug-RNA interface. Our studies demonstrate that it is possible to detect a drug-sized molecule interacting with the RNA, though we cannot show the atomic structure detail because of the limited resolution. Our B-factor plot in Supplementary Figure 2d indicates that our resolution is limited by the number of particle images and their inherently low contrast. This can be improved with more particle images and/or possibly with improvement in the instrument (e.g. better DQE of next generation of the detector and better phase plate), which suggests the promise of our approach and the future direction of this project.

Major Comments/Suggestions:

1. What's the local resolution of the ligand-binding pocket in the apo and SAM-bound structure? Maybe it's better to show an expanded view of it.
Response: We thank the reviewer for this important question and suggestion. We have updated Supplementary Figure 2f and Supplementary Figure 3f to show the expanded view of the ligand-binding pocket. The local resolution is around 4.5 Å based on Resolution map.

2. In the discussion, it was suggested to relate the current cryo-EM structural results of SAM-IV riboswitch with the previous functional study such as the results in the reference [Weinberg, Z. et al. RNA 14, 822-828 (2008)] if the authors are not planning to start other validation experiments.
Response: In the reference, it is noted that "SAM-IV riboswitches do not conserve the nucleotide corresponding to U88 in SAM-I". Our model of the ligand is based on the SAM-I structure (PDB: 3GX5), however, it may not be possible to further relate the two structures without further functional studies as the reviewer mentions. The structural information presented here will be a good basis for such studies in the future.

3. In Supplementary Figure 2a, what's the difference between the refinement result of Relion and CryoSparc? How to improve the resolution from 3.93 Å to 3.7 Å? Is it possible to align them together and give a comparison?
Response: The results from Relion and CryoSPARC have the same overall shape with consistent features. The comparison is shown in Supplementary Figure 2b with the cross-correlation coefficient of 0.96. During the data processing, we tried several different software packages to try to get a better map. We found that the new options of non-uniform refinement and local

refinement in CryoSPARC version 2 facilitated the acquisition of higher-resolution structures for some dynamic or flexible specimens. So in this work, we tried the options and improved the resolution by 0.2 Å to achieve slightly better structural features.

We have edited the Methods to clarify our protocol that the final refinements in Relion and CryoSPARC were performed separately, not sequentially, on page 10. Their similarity reinforced the convergence of the structure. Thus, we include a supplement figure (Supplementary Figure 2b) to substantiate our claim.

4. In the panel d of Supplementary Figure 3, it seems the peripheral resolution is lower than 5 Å. But in Page 6 line 115, it was mentioned that "the kink-turn motif between P2a and P2b helices in the SAM-I is substituted for a distinct turn in SAM-IV". How did you define the "distinct turn"?
Response: We thank the reviewer for pointing this out. We reached this conclusion based on models. The kink-turn motif in SAM-I consists of six nucleotides, while the turn in SAM-IV has only four nucleotides, and has a backbone path unlike the kink in the other structure, so we called it a distinct turn. We added this on page 6 to clarify.

5. In Supplementary Figure 6c, it seems the Mean.Q (3.7 Å)=0.46 curve is below the average position of the Entire NT, Backbone and Base curves. Please check them.
Response: The "Mean Q" that is illustrated by the horizontal lines in these figures reflects the expected Q-score at this resolution based on Q-scores of 52 maps & models of RNA structures from the EMDB database (Pintilie, G.. doi:10.1101/722991). We have now relabeled it as "Expected Q" to be more accurate. The fact that most of our per-residue Q-scores are higher than this line indicates that our structures have better Q-scores than other reported cryo-EM RNA-containing structures at similar resolution.

Minor Comments/Suggestions:

1. Page 2, line 38, change "possible" to "potential".
Response: Done.

2. Since there is enough space in the Supplementary Figure 8, the topology figure and the tertiary figure of three riboswitches in each panel can be shown side to side to make the view larger.
Response: Done.

3. In stem P5, do A86 and G104 form the non-canonical base pair? How about the terminal bases G81 and A109?
Response: We thank this reviewer for the insightful question. Residues G81 and A109, A86 and G104 form non-canonical base pairs in some, but not all of the auto-DRRAFTER models. At the

current estimated accuracy level (3.5-3.6Å, estimated from auto-DRRAFTER modeling convergence, see “Kappel, K. et al. doi:10.1101/717801” for details), we cannot confidently conclude whether these base pairs are forming. We note, however, that the base pairs shown in the secondary structure diagram in Fig. 2a are consistent with the results of M2-seq experiments described in this reference (Kappel, K. et al. doi:10.1101/717801).

4. The Abstract in Nature Communications was requested not to contain references, please check the format of the abstract.
Response: Done.

5. Page 7, line 155, change “and” to “or”.
Response: Done.

6. Page 7, line 158, “RNA was prepared as describe in 13”. It seems the authors missed the word “reference”.
Response: Done.

REVIEWERS' COMMENTS:

Reviewer #2 (Remarks to the Author):

The authors have addressed all the reviewers' questions. I recommend to accept this work as it is right now.

-Hongwei Wang

Reviewer #3 (Remarks to the Author):

During revision, the manuscript "Cryo-EM Structure of a 40-kDa SAM-IV Riboswitch RNA at 3.7 Å Resolution" by Zhang, K. et al. that reports the structures of apo and SAM-bound SAM-IV riboswitches was improved substantlly. Most of the reviewers' concerns have been addressed. I recommend the publication of this work with minor improvement.

1. In Fig.2a (the secondary structure of SAM-VI riboswitch), some residues such as U10, G17, U31, G111 and C112 are too close to other adjacent residues, please seperate them.

2. In Fig. 3, according to the computation, the location of the ligand was confirmed. However, it seemed the adenine moiety in Fig. 3d didn't fit the wire frame density well, how to confirm the local conformation of the ligand? Is it possible for the adenine moiety to rotate in the binding pocket and fit the density?

3. The authors should pay more attention to the writing and the format of Nature. Commu.

Responses to the reviewers' comments on Manuscript entitled "Cryo-EM Structure of a 40-kDa SAM-IV Riboswitch RNA at 3.7 Å Resolution"

Reviewer #2 (Remarks to the Author):

The authors have addressed all the reviewers' questions. I recommend to accept this work as it is right now.

-Hongwei Wang

Reviewer #3 (Remarks to the Author):

During revision, the manuscript "Cryo-EM Structure of a 40-kDa SAM-IV Riboswitch RNA at 3.7 Å Resolution" by Zhang, K. et al. that reports the structures of apo and SAM-bound SAM-IV riboswitches was improved substantlly. Most of the reviewers' concerns have been addressed. I recommend the publication of this work with minor improvement.

1. In Fig.2a (the secondary structure of SAM-VI riboswitch), some residues such as U10, G17, U31, G111 and C112 are too close to other adjacent residues, please seperate them.
Response: We thank the reviewer for pointing this out. We have revised the Fig. 2a.

2. In Fig. 3, according to the computation, the location of the ligand was confirmed. However, it seemed the adenine moiety in Fig. 3d didn't fit the wire frame density well, how to confirm the local conformation of the ligand? Is it possible for the adenine moiety to rotate in the binding pocket and fit the density?

Response: We thank the reviewer for these questions. We have undertaken a series of analysis to confirm the location of the ligand and demonstrated that it is possible to detect a drug-sized molecule interacting with the RNA, though we cannot show the atomic structure detail as well as confirm its conformation because of the limited resolution. Actually, our model of the ligand is

based on the SAM-I structure (PDB: 3GX5), including its conformation. In Fig. 3d, a separate density peak in an automatically segmented map was identified as the position of the ligand using *Segger* (Pintilie, G. et al. J. Struct. Biol. 170, 427–438 (2010)), fitted with the ligand model derived from SAM-I crystal structure (PDB code: 3GX5). Compared with Fig. 3c, the elongated density below the adenine moiety in Fig. 3d may be caused by the limited resolution, resulting in the difference in extracted density between different methods. To avoid bias, although the adenine moiety didn't fit the wire frame density well, we did not refine or rotate the ligand in the binding pocket. However, the Q-scores of adenine, sulfonium, and aminoacyl group, three moieties of the ligand model, are 0.38, 0.35, and 0.29, respectively (Fig. 3d), which are comparable to the expected Q-score (0.40) of ribosomal RNA models at 4.1-Å resolution (Supplementary Figure 6c, d), indicating the reliability of SAM positioning in our cryo-EM map.

3. The authors should pay more attention to the writing and the format of Nature. Commu.

Response: The reviewer's comment is important and well taken. We have revised the whole manuscript carefully.
